# Small-scale transcriptomics reveals differences among gonadal stages in Asian seabass (*Lates calcarifer*)

**DOI:** 10.1186/1477-7827-12-5

**Published:** 2014-01-09

**Authors:** Preethi Ravi, Junhui Jiang, Woei Chang Liew, László Orbán

**Affiliations:** 1 Reproductive Genomics Group, Temasek Life Sciences Laboratory, 1 Research Link National University of Singapore, Singapore 117604, Singapore; 2 Department of Biological Sciences, National University of Singapore, 21 Lower Kent Ridge Rd, Singapore 119077, Singapore; 3 Agri-Food and Veterinary Authority of Singapore, 5 Maxwell Rd, Singapore 069110, Singapore; 4 School of Biological Sciences, Nanyang Technological University, 50 Nanyang Ave, Singapore 639798, Singapore; 5 Department of Animal Sciences and Animal Husbandry, Georgikon Faculty, University of Pannonia, H-8360, Keszthely, Hungary; 6 Present address: National Centre for Biological Sciences, Tata Institute of Fundamental Research, GKVK, Bellary Road, Bangalore 560065, India

**Keywords:** qPCR, Barramundi, Maturation, Sex, Gene expression

## Abstract

**Background:**

The Asian seabass *(Lates calcarifer)* is a protandrous hermaphrodite that typically matures as a male at approximately 2–4 years of age and then changes sex in subsequent years. Although several sexual maturation stages have been described histologically for both testis and ovary, the underlying gene expression profiles remain lacking. The development of a gene expression platform is therefore necessary to improve our understanding of the gonad development of this cultured teleost species.

**Methods:**

Thirty Asian seabass gonads were collected from farms in Singapore, examined histologically and staged according to their sex and gonadal maturation status. Partial coding sequences of 24 sex-related genes were cloned using degenerate primers and were sequenced. Additional 13 cDNA sequences were obtained through next-generation sequencing. A real-time qPCR was then performed using the microfluidic-based Fluidigm 48.48 Dynamic arrays.

**Results:**

We obtained 17 ovaries and 13 testes at various stages of sexual maturation. Of the 37 genes that were tested, 32 (86%) showed sexually dimorphic expression. These genes included sex-related genes, *sox9*, *wt1*, *amh*, *nr5a2*, *dmrt1* and *nr0b1*, which showed testis-enhanced expression similar to other vertebrate species. Known male- and female-enhanced germ cells markers, which were established from studies in other species, similarly showed testis- and ovary-enhanced expression, respectively, in the Asian seabass. Three pro-Wnt signaling genes were also upregulated in the ovary, consistent with existing studies that suggested the role of Wnt signaling in ovarian differentiation in teleosts and mammals. The expression patterns of genes involved in steroidogenesis, retinoic acid metabolism, apoptosis and NF-κB signaling were also described. We were able to classify gonads according to sex and gonadal maturation stages, based on their small-scale transcriptomic profiles, and to uncover a wide variation in expression profiles among individuals of the same sex.

**Conclusions:**

The analysis of a selected set of genes related to reproduction and in sufficient number of individuals using a qPCR array can elucidate new insights into the molecular mechanisms involved in Asian seabass gonad development. Given the conservation of gene expression patterns found in this study, these insights may also help us draw parallels with other teleosts.

## Background

The Asian seabass (*Lates calcarifer*), which is also commonly known as barramundi, is a protandrous hermaphrodite. Typically, this perciform teleost matures as a functional male at 2–4 years of age and subsequently changes sex to female at subsequent spawning seasons [1-3]. Due to this process, Asian seabass females are generally larger than males.

Primary females that do not appear to go through the male phase, as well as males that do not undergo sex change, may also exist, and these possibilities have been inferred from the presence of young, small-sized females and old males, respectively [1,3]. In addition, despite the late sexual maturation age of the species, early testis differentiation can be found in Asian seabass that are grown in intensive freshwater recirculation systems at as early as 9 months old [4].

Although the development of Asian seabass gonads has been well studied morphologically and histologically, the underlying molecular data regarding their development is lacking [1-4]. This lack is despite the identification of several Asian seabass orthologs from the NCBI nucleotide database, including the ovarian aromatase, follistatin and vitellogenin, which are conserved in several teleost species. Next-generation sequencing technologies have only been recently used to provide functional genomics analyses of other aspects of the Asian seabass biology, such as temperature adaptation and the response to stress [5,6].

To date, several important genes have been found to be required for testis or ovary differentiation in a number of model organisms. These sex-related genes include *sox9*, *wt1*, *amh*, *nr5a2*, *dmrt1* and *nr0b1*, which produce proteins that are required for testis differentiation and have enhanced expression in the testis of several vertebrate species (see Additional file 1: Table S1 for full gene names) [7-13]. For ovarian differentiation, several studies have suggested the role of Wnt signaling in both mammals and teleosts [14-17].

Genes that are involved in steroidogenesis also play a key role in gonad differentiation because these genes lead to the production of sex steroids. Several studies have shown that artificial sex reversal can be easily induced in teleosts through the application of hormones or endocrine-disruptors [18,19]. Several steroidogenic genes, such as the ovarian aromatase (*cyp19a1*) and 11ß-hydroxgenase (*cyp11c1*), tend to show sexually dimorphic expression in teleosts [9,20-23]. Other than sex steroids, recent studies of the Japanese flounder (*Paralichthys olivaceus*) and pejerrey (*Odontesthes bonariensis*) have also shown that cortisol may (a steroid hormone) have a role in testis differentiation [24,25].

Germ cells are key components of the gonads, and their numbers in the early teleost gonads have been suggested to affect sex determination [26]. Several germ cell markers, such as *piwil1* in the zebrafish *(Danio rerio)* and *sycp* in the medaka *(Oryzias latipes)*, displayed sexually dimorphic expression in the gonads [27,28]. This observation could be due to the structural functions that some germ cell markers have in specific germ cell types, such the role of ODF proteins in forming the cytoskeletal structure of the human sperm tail [29]. Germ cell markers can also play other non-structural roles, such as sperm receptors (*zona pellucida* genes) or the maintenance of transposon silencing in the germline (*piwi-like* genes) [30,31]. In addition to key sex-related genes, steroidogenic genes, Wnt signaling genes and germ cell markers, other genes that are involved in retinoic acid signaling, apoptosis and NF-κB signaling have also been implicated in teleost sex differentiation [32,33].

Based on our current state of knowledge regarding sex differentiation, we shortlisted genes that are involved in the pathways or functions that have been described above for gene expression analysis. In this study, we generated a set of real-time qPCR primers for 37 genes and performed gene expression profiling of Asian seabass testes and ovaries on a mid-throughput microfluidic-based qPCR platform. Our analysis showed that several genes with known reproductive function(s) (e.g., Wnt pathway genes and germ cell markers) exhibited expression patterns in the Asian seabass that were similar to those of other teleosts. We were also able to classify gonads according to sexual maturation status by hierarchical clustering analysis using expression profiles of these genes. In addition, the expression analysis revealed interesting aspects of the Asian seabass gonads and uncovered new insights into their sexual maturation.

## Methods

### Fish

Adult Asian seabass individuals that ranged from 44 – 65 cm standard length were collected from the Marine Aquaculture Centre of the Agri-Food and Veterinary Authority of Singapore. The fish were reared and maintained according to protocols that were approved by the AVA Institutional Animal Care and Use Committee (approval number: AVA-MAC-2011-01). The fish were sacrificed, and gonads were collected for histology and RNA extraction.

### RNA isolation and cDNA synthesis

Total RNA was extracted from the gonads using the RNeasy Mini Kit (Qiagen). For the real-time qPCR experiment, RNA quality and quantity were assessed using agarose gel electrophoresis and a NanoDrop spectrophotometer, respectively. Total RNAs were reverse transcribed using an iScript cDNA Synthesis Kit (Bio-Rad Laboratories) following the manufacturer’s instructions.

### Histology and staging of gonads

Gonads were fixed in 10% formalin overnight at room temperature. After dehydration by ethanol, samples were embedded in plastic resin (Leica Biosystems), and then serial cross-sections of 5–10 μm were cut using a microtome (Leica Biosystems) and dried on slides at 42°C overnight. The sections were stained with hematoxylin and eosin and then mounted in Permount (Thermo Fisher Scientific).

The Asian seabass gonads were classified according to sexual maturation status as described by Guiguen and colleagues [2]. We obtained 17 ovaries, with four at the F1 stage, seven at the F3 stage and six at the F4 stage, as well as 13 testes, with three at the M1 stage, two at the M2 stage, six at the M3 stage and two at the M4 stage (Table 1, see Additional file 2: Figure S1 for the gonad histology).

**Table 1:** Classification of gonadal samples based on histology

**Sex type**	**Index**	**Gonadal maturation stage**	**Histological criterion**	**Number of samples**
***Female***	F1	Pre-vitellogenesis	Mostly pre-vitellogenic oocytes	4
	F3	Vitellogenesis	Vitellogenic oocytes more than half of section	7
	F4	Atretic	Presence of atretic oocytes	6
***Male***	M1	Testis gonia	Mostly gonia	3
	M2	Spermatogenesis	Mostly spermatocytes and spermatids	2
	M3	Spermiation	Mostly spermatozoa	6
	M4	Post-spawning	Absence of spermatozoa in testicular lobules	2
		**Total number of samples**		**30**

### Isolation, cloning and sequencing of genes with sex-related function

Partial coding sequence of Asian seabass genes with sex-related function were obtained using degenerate primer-based amplification and cloning. Several known homologous cDNAs from other teleost species were clustered using ClustalW, and degenerate primers were designed at conserved regions using the software Primer Premier v5.0 (Premier Biosoft) [34]. PCR was then performed on pooled cDNA, which originated from Asian seabass testes and ovaries. The degenerate primer sequences and annealing temperatures that were used for PCR are listed in Additional file 3: Table S2. The PCR products were run on a 2% agarose gel and stained using GelStar nucleic acid gel stain (Lonza). The band of the expected size was cut from the gel, and then the amplified product was isolated using the QIAquick Gel Extraction Kit (Qiagen) and cloned into the pGEM-T Easy Vector (Promega). The validity of the sequence and the identification of the Asian seabass orthologous gene were completed by running the BLAST algorithm (blastn) against the NCBI RefSeq database (Additional file 1: Table S1).

### Transcriptome sequencing

Total RNAs, which were obtained from Asian seabass testes and ovaries, were depleted of ribosomal RNA using a RiboMinus Eukaryote Kit for RNA-seq (Invitrogen) and verified using an Agilent 2100 Bioanalyzer. The rRNA-depleted total RNA was sent to a service provider for transcriptome sequencing on a SOLiD 3+ platform (Applied Biosystems). Similarly, another rRNA-depleted sample, which was pooled from RNA that was extracted from various Asian seabass organs, was sent to another service provider for transcriptome sequencing on a 454 FLX Titanium platform (Roche). Reads that were obtained from SOLiD 3+ and 454 sequencing were assembled *de novo* using the programs Velvet, CLC Genomics Workbench (CLC Bio) and Sequencher (Gene Codes) [35]. The reads and assembled transcriptome have been deposited into the NCBI SRA and TSA databases, respectively [SRA accession numbers SRR944005, SRR944006 and SRR949061; TSA accession numbers GAML01000000 and GAMU01000000].

### Real-time qPCR

Real-time qPCR was performed using a BioMark^TM^ HD system (Fluidigm Corporation). cDNA from Asian seabass testes and ovaries were used for specific target amplification using the TaqMan PreAmp Master Mix (Life Technologies) and loaded onto Fluidigm’s Dynamic Array Integrated Fluidic Circuits (IFC) according to Fluidigm’s EvaGreen DNA Binding Dye protocols. The gene symbols, their corresponding accession numbers and gene names are listed in Additional file 1: Table S1, and the primer sequences that were used are listed in Additional file 4: Table S3.

Four 48.48 Dynamic Array IFC plates were used to analyze the expression levels of selected genes in Asian seabass testes and ovaries. Triplicates were analyzed for each biological sample, and four samples were used as inter-plate controls.

Eight genes (*18 s*, *bactin*, *ef1a*, *gapdh*, *rpl8*, *tuba*, *catD* and *ubq*) were included into the qPCR array to select for endogenous reference genes using the geNorm algorithm [36]. Of these genes, *rpl8*, *ef1a* and *ubq* had the highest gene expression stability and were used as reference genes (see Additional file 5: Figure S2 for the stability values).

## Results and discussion

### The vast majority of genes showed sexually dimorphic gene expression pattern between male and female gonads

Several genes, which have important reproductive functions in mammals and other vertebrates, have been included in this qPCR array. Of the 37 genes chosen in this study (not including the eight reference genes), 86% (32/37) of these genes showed sexually dimorphic expression (p-value < 0.05 and fold-change ≥ 1.5 or ≤ -1.5) when ovaries of F3 and F4 stages were compared against testes of M3 and M4 stages (Table 2). The expression of 25 of these genes was enhanced in testes, with 13 genes showing more than 10-fold upregulation when compared with ovaries. In contrast, seven genes were found to have ovary-enhanced expression, of which *zp2* showed the highest upregulation (7.1-fold) in ovaries compared with testes.

**Table 2:** Genes that were analyzed between Asian seabass testes (M3 and M4) and ovaries (F3 and F4) and classified according to functions and pathways

**Gene symbol**	**Accession**	**Fold-change (Ovary vs. Testis)**	**p-value**
***Known sex-related***			
*sox9*	KF444460	-51.8	4.95E-04
*wt1*	KF444464	-25.0	0.024
*amh*	GAMU01071817	-19.7	9.49E-10
*nr5a2*	KF444453	-15.2	0.007
*dmrt1*	KF444450	-7.8	0.000
*nr0b1*	KF444458	-7.6	0.013
***Germ cell markers***			
*odf3*	GAMU01119126	-2628.1	2.64E-13
*sycp3l*	GAML01036838	-25.8	1.16E-06
*tdrd7*	GAML01004579	-15.4	1.37E-08
*piwil1*	GAML01007253	-10.6	4.26E-10
*sept6*	KF444459	-7.5	2.27E-10
*vasa*	KF444462	-3.2	1.18E-04
*tdrd1*	GAML01005267	-2.2	0.002
*sept7*	GAML01001618	-1.5	0.026
*zp2*	KF444465	7.1	0.003
***Steroidogenesis***			
*cyp11c1*	KF444447	-377.8	1.56E-15
*hsd3b*	KF444455	-42.1	0.038
*hsd11b2*	KF444456	-12.8	0.025
*cyp19a1*	AY684256.1	-5.5	1.47E-04
*cyp17a1*	KF444448	-5.0	0.005
*hsd17b1*	KF444457	-4.2	0.786 (N.S.)
***Wnt signaling***			
*foxl2*	KF444454	-4.9	0.434 (N.S.)
*axin1*	KF444443	-3.1	0.242 (N.S.)
*ctnnbip1*	KF444446	1.5	0.072 (N.S.)
*ck2a*	KF444444	2.0	0.042
*ctnnb1*	KF444445	3.5	0.001
*dvl2*	KF444451	4.5	0.001
***Retinoic acid signaling***			
*cyp26b1*	KF444449	-36.3	5.61E-06
*stra6*	GAML01004693	1.3	0.079 (N.S.)
*cyp26a1*	GAML01005182	1.8	0.030
			
***NF-κB-related***			
*nfkb2*	GAMU01013914	-3.1	2.74E-04
*nkap*	GAML01003947	-3.0	0.001
***Apoptosis***			
*tp53*	KF444461	2.0	0.020
***Others***			
*esr1*	KF444452	-25.1	6.68E-05
*ar*	KF444442	-4.9	2.29E-06
*psap*	GAML01003564	-2.1	0.001
*vtgr*	KF444463	2.9	0.009

N.S. - Not significant (fold-change < 1.5 or > -1.5 or p-value ≥ 0.05).

### Conserved expression patterns of sex-related genes and pathways in the Asian seabass with other teleosts and even mammals

#### Known sex-related genes

Among the genes in the array, there were genes with “pro-male” functions, such as *amh, sox9*, *dmrt1*, *wt1*, *nr5a2* and *nr0b1*, which displayed conservation of testis-enhanced expression in the Asian seabass that was similar to other vertebrates (Table 2). For example, in humans, anti-Müllerian hormone (AMH), which is produced by fetal Sertoli cells, is responsible for the regression of Müllerian ducts during testis differentiation, and its mutation results in the presence of uteri and Fallopian tubes in males [37]. Amh also inhibits follicular growth and aromatase expression in mouse ovaries [38,39]. In teleosts, *amh* displayed a higher expression in zebrafish testes than in ovaries, and likewise, we detected a strong testis-enhanced expression of *amh* (19.7-fold upregulation) in the Asian seabass [9,21]. Similarly, *sox9*, *dmrt1*, *wt1*, *nr5a2* and *nr0b1* were also found to have increased expression in Asian seabass testes compared with ovaries, and earlier, their orthologs were shown to have roles in testis development in other teleosts and even in mammals [7-13].

#### Germ cell markers

We have also included several germ cell markers in this array. One of these markers, *piwil1*, has a role in maintaining transposon silencing in the germline genome and has higher expression in testes of adult zebrafish compared with the ovaries [27,30]. Similarly, *piwil1* was found to have higher expression in Asian seabass testes (Table 2). Another set of germ cell markers, Tudor domain containing proteins or Tdrds, are associated with the nuage or germinal granules of germ cells and have been found to be highly expressed in adult mouse testes [40]. In our array, *tdrd1* and *tdrd7* also showed testis-enhanced expression in the Asian seabass.

The other four germ cell markers, *odf3*, *sycp3l*, *sept6* and *sept7*, are known to be expressed in the spermatids and spermatozoa of other teleost and mammalian species. ODF proteins form the main cytoskeletal structure of the human sperm tail and are found to be essential for male fertility in the zebrafish [29,41]. Because of this structural function, *odf3* expression levels had the highest fold difference (2,628-fold) in Asian seabass testes compared with ovaries (Table 2). SCP3 is essential in meiosis progression and in the formation of spermatozoa in mice [42]. In the medaka, *sycp* has been shown to have higher expression in testes than ovaries, and likewise, we detected a 25.8-fold upregulation in Asian seabass testes (Table 2) [28]. Septins are a family of GTP-binding proteins that forms a component of the cytoskeleton in eukaryotes [43]. These proteins have been shown to be required for sperm development in mouse and have sexually dimorphic expression in zebrafish gonads [22,44]. In Asian seabass, *sept6* and *sept7* have 7.5-fold and 1.5-fold higher expression in testes compared with ovaries, respectively (Table 2). Additionally, the germ cell markers that we have investigated have displayed conservation in gene expression patterns with other vertebrate species.

#### Steroidogenesis-related genes

In teleosts, 17β-estradiol (E2) and 11*-*ketotestosterone (11-KT) are the key female and male hormones, respectively, and sex differentiation can be easily influenced by steroids or endocrine-disruptors, which result in sex reversal [18,19,45,46]. Both E2 and 11-KT are synthesized from testosterone, and *cyp11c1* converts testosterone to 11-KT [45]. Therefore, *cyp11c1* has an important role in testicular development, and this role is reflected in its testis-enhanced expression in teleosts, including the European seabass (*Dicentrarchus labrax*) and zebrafish [20,22]. Accordingly, in the Asian seabass, *cyp11c1* has over 300-fold upregulation in the testis (Table 2).

In contrast, *cyp19a1a*, or ovarian aromatase, is involved in the conversion of testosterone to E2. During zebrafish gonadal transformation from juvenile ovaries to testes, *cyp19a1a* is downregulated, whereas its expression remains high in adult ovaries [9,21,23]. The overexpression of aromatase can also result in ovary development in genetically male chicken embryos [47]. However, contrary to our expectation, an upregulation of gonadal aromatase was found in Asian seabass testes compared with ovaries (Table 2). One possible explanation is that, although *cyp19a1* was overexpressed by 5.5-fold in the testis, *cyp11c1*, which is the gene that regulates the 11-KT level, was overexpressed by more than 300-fold. Given that both *cyp19a1* and *cyp11b* act on the same precursor, which is testosterone, this result may indicate that estrogen levels may remain low relative to those levels of androgens in males. In addition, Cyp19a1 may also be regulated at the post-transcriptional level in the seabass gonads. It was also recently found that, in some cichlid lineages, both the ovarian and brain aromatases can have testicular function, and as a result, the sex steroid pathway has been suggested to be less conserved among teleosts [48]. Nevertheless, the unexpected testis-enhanced expression of *cyp19a1* in the Asian seabass is worth further investigation in the future.

In recent years, cortisol has been shown to be involved in teleost sex differentiation. For example, in the Japanese flounder (*Paralichthys olivaceus*) and in pejerrey (*Odontesthes bonariensis*), elevated cortisol levels that are caused by increased temperature can result in female-to-male sex reversal [24,25]. Therefore, in this qPCR array, we have also included two genes that are involved in the metabolism of cortisol, which is also an important component of the stress response in fish [49]. Although the interrenal tissues of the head kidney are known to be the major site of cortisol production in teleosts, it is, nevertheless, worthwhile to investigate the gonadal expression of the two genes *hsd11b2* and *hsd3b*[50].

Hsd11b2 plays a role in the conversion of the physiologically active cortisol to inactive cortisone [51]. It has been suggested that 11ß-hydroxysteroid dehydrogenases protect the teleost gonads from the inhibitory effects of cortisol, such as the inhibition of testicular androgen production [52,53]. In contrast, *hsd3b* is involved in the early steps of the steroidogenic pathway, which results in the production of not only glucocorticoids but also mineralocorticoids and sex steroids [54]. The *hsd3b* gene was also found to be expressed in both the interrenal tissues of the head kidney and in the gonads of zebrafish [54].

In the Asian seabass, both genes were overexpressed in testes compared with ovaries. The gene of the cortisol-producing enzyme *hsd3b* displayed over 40-fold upregulation, whereas the gene of the cortisol-degrading enzyme, *hsd11b2*, exhibited 12.8-fold overexpression (Table 2). Therefore, cortisol might also have reproduction-related roles in the Asian seabass because of the sexually dimorphic expression of both genes.

#### Wnt signaling pathway

Canonical Wnt signaling has been known to be involved in mammalian ovary development, and the overexpression of WNT4 in humans has been associated with XY sex reversal [15,16,55]. Similarly, Wnt signaling has already been implicated in a reproductive role in teleosts, including the black porgy (*Acanthopagrus schlegeli*), rainbow trout (*Oncorhynchus mykiss*) and zebrafish [14,17,56,57]. It has been further shown that Wnt signaling could promote ovarian differentiation through the upregulation of gonadal aromatase [56,58].

Among the sexually dimorphic genes in the Asian seabass, there were three members of the Wnt family: *β-catenin 1 (ctnnb1)*, *dvl2* and *ck2a*. β-catenin is the central molecule in the canonical Wnt signaling pathway. In the absence of Wnt ligand binding, cytoplasmic β-catenin is degraded by the ubiquitin-proteosome pathway and prevented from entering the nucleus and associating with *Lef1* to activate Wnt target genes [59,60]. *Dishevelled* is a positive transducer of Wnt signals and functions to activate both canonical and non-canonical Wnt signaling pathways [61]. In mouse, *Ck2* phosphorylates β-catenin and, therefore, protects β-catenin from being degraded by the ubiquitin-proteosome pathway [62]. *Ck2* has also been found to be activated by Wnt3a [63]. All three “pro-Wnt signaling” genes were upregulated in Asian seabass ovaries compared with testes, and this result suggested that, similar to mammals and other teleost species, the Wnt signaling pathway has a pro-female function in this species as well (Table 2).

#### Retinoic acid (RA) signaling pathway

RA is catabolized by both *Cyp26a1* and *Cyp26b1*[64]. In mice, *Cyp26b1* is required to retard or block germ cells from entry into meiosis in the testes and to prevent the apoptosis or conversion of the male germ cell fate [65,66]. *Cyp26b1* expression in mice is also thought to be activated by Sox9 and Sf1 in the testes and inhibited by Foxl2 in the ovaries [67]. Although little is known regarding the role of *Cyp26a1* in reproduction, the exposure of mice to RA resulted in the apoptosis of spermatogonia and in the increased expression of this gene [68].

In the Asian seabass, *cyp26b1* was strongly upregulated in testes compared with ovaries, whereas there was only a slight downregulation of *cyp26a1* (Table 2). Thus, the sexually dimorphic expression of *cyp26b1* and *cyp26a1* suggest the possibility of retinoic acid signaling involvement in testes development in the Asian seabass.

#### Genes with apoptosis-related function

During gonadal transformation in zebrafish, apoptosis is required to remove the unwanted female cells to ‘clear up space’ for the developing testicular cells [69,70]. In zebrafish, the main function of *fancl* is to ensure the survival of female germ cells. As a result, its mutation causes zebrafish to develop as males, and its effects can be rescued through the mutation of the *tp53* tumor suppressor gene, which is a well-known pro-apoptotic gene [71,72]. Our results demonstrated that *tp53* was upregulated by two-fold in Asian seabass ovaries compared with testes. It is possible that this result is due to the atresia that occurs in the F4 ovaries and that the testes that are not undergoing transformation to ovaries are unlikely to have higher levels of apoptosis.

#### NF-κB pathway

NF-κB is a ubiquitous transcription factor that has been known to be involved in several biological processes, which include the immune response [73-75]. In mammals, NF-κB was shown to interact with SF-1 and to prevent SF-1 from activating Amh, thus inhibiting Amh expression [76]. In the zebrafish, NF-κB has been shown to inhibit apoptosis during gonad transformation, and thereby, to promote female bias [77]. In contrast, NKAP is an activator of NF-κB, is also shown to repress Notch signaling and is necessary for T-cell development [78,79]. Although the NF-κB pathway seems to be a “pro-female” pathway, both genes (i.e., *nfkb2* and *nkap*) were downregulated in Asian seabass ovaries compared with testes. In the adult gonads of Asian seabass, *NF-κB* may have additional reproduction-related roles as suggested by the sexually dimorphic expressions of *nfkb2* and *nkap*.

### The expression profiles of 36 genes were sufficient to distinguish between male and female gonad types in the Asian seabass

**Figure 1:**
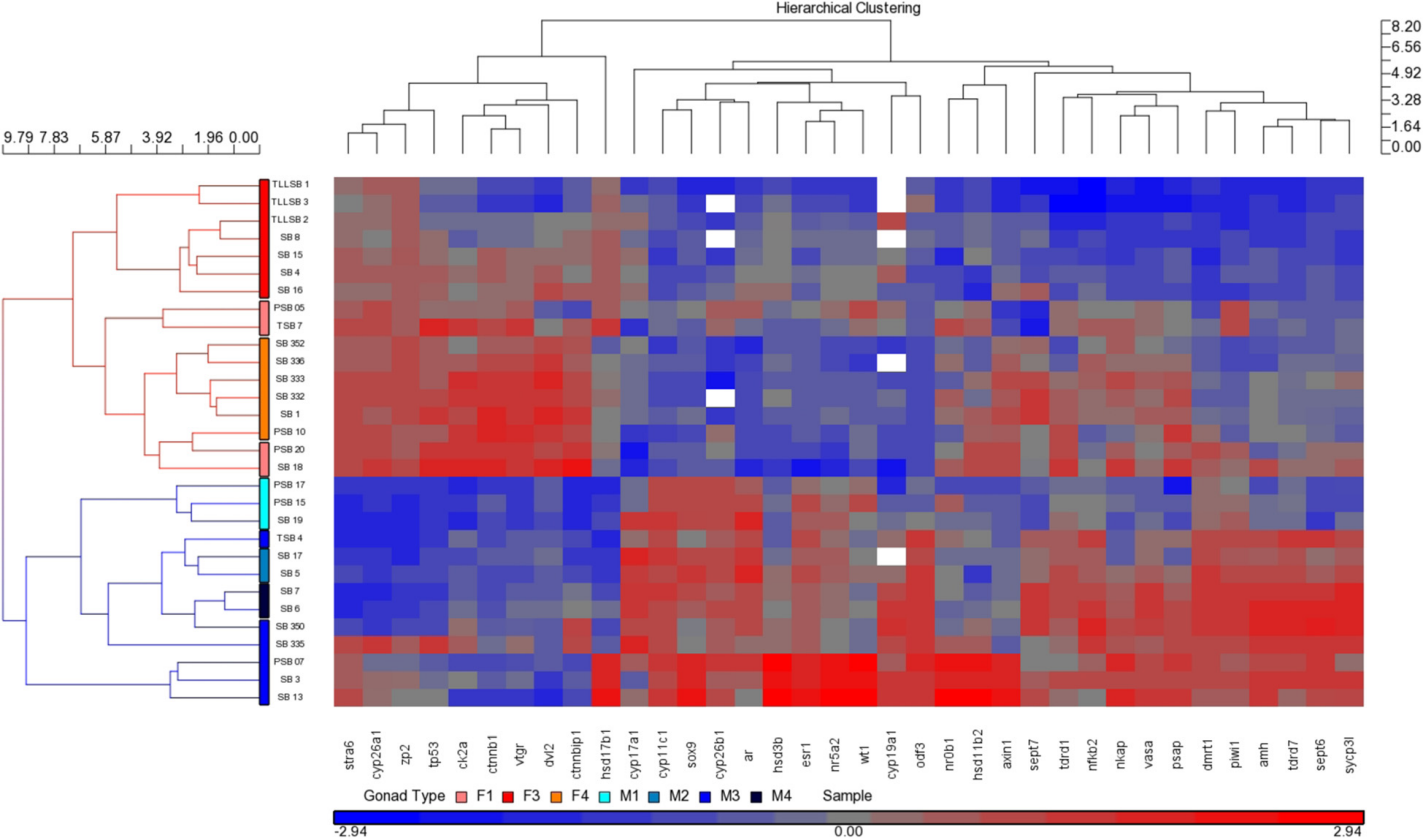
**Separation of the male and female Asian seabass gonads under the hierarchical clustering map.** The expression profiles of 36 sex-related genes with p-value (gonad type) < 0.01 under a one-way ANOVA analysis were used to generate the hierarchical clustering map. Male and female gonads were clustered into two different clades. Within the female gonad clade (top section), F3 and F4 ovaries were further divided into two sub-clades, which indicated that their gene expression profiles were different. Red boxes indicate high expression, whereas blue boxes indicate low expression. White boxes within the clustering map indicate missing values for a particular sample and gene.

A one-way ANOVA was performed, and all the genes that were tested, except *foxl2* and the reference genes, showed significant differences in their expression levels across the various gonad types (p-value < 0.05). The expression profiles of these 36 genes were used to generate a hierarchical clustering map using the Partek Genomics v6 Suite (Partek Inc.) software (Figure 1).

From the hierarchical clustering map, we can observe that the ovaries and testes samples were clustered into two separate primary clades, and within the ovary clade, the F3 ovaries were clustered into their own sub-clade (Figure 1). This result indicated that the F3 ovaries had a distinct gene expression profile from that of ovaries from other stages. In particular, although F3 and F4 ovaries looked similar under histology, 70% of the tested genes (26/37) were differentially expressed between these two stages.

The genes that were upregulated in F4 ovaries compared with F3 ovaries included germ cell markers, such as *vasa*, *piwil1*, *tdrd1* and *sycp3l*; testis-enhanced genes, such as *dmrt1*, *amh*, *nr0b1*; Wnt signaling member genes, such as *axin1*, *dvl2*, *ctnnb1*, *ck2a* and *ctnnbip1*; apoptosis-related genes, such as *tp53* and genes that are involved in the retinoic acid pathway (*cyp26a1* and *stra6*) (Additional file 6: Table S4). The key histological feature that separates F3 and F4 ovaries were the presence of atretic oocytes (Additional file 2: Figure S1). The pro-apoptotic gene *tp53* was upregulated by 2.6-fold in the F4 ovary compared with the F3 ovary, and this upregulation may be a consequence of atresia in the F4 ovary. In addition, the upregulation of 24 genes and the downregulation of only three genes in F4 ovaries compared with F3 ovaries suggest that the former may be more transcriptomically active than the latter.

We can also observe from the hierarchical clustering map that individual differences in gene expression exist within each group, which shows the complexity and variability in the process of gonad development. Studies regarding gonad differentiation in the zebrafish have also reported such a trend [80,81]. The individual variability of gonad development hence dictates that more biological samples would be required to obtain a more representative result for any sex-related experiment. In this sense, the use of a 48.48-type qPCR array would prove useful because this method allows the analysis of only the important genes in several individuals (up to 13) in parallel. This result also demonstrated that the analysis of a moderate number of well-selected genes that are relevant to the area of interest (reproduction in our case) and in a sufficient number of individuals could be a powerful tool for improving our understanding of the molecular regulation of these complex processes.

### Female-like expression levels of *amh* and germ cell markers in M1 testes

**Figure 2:**
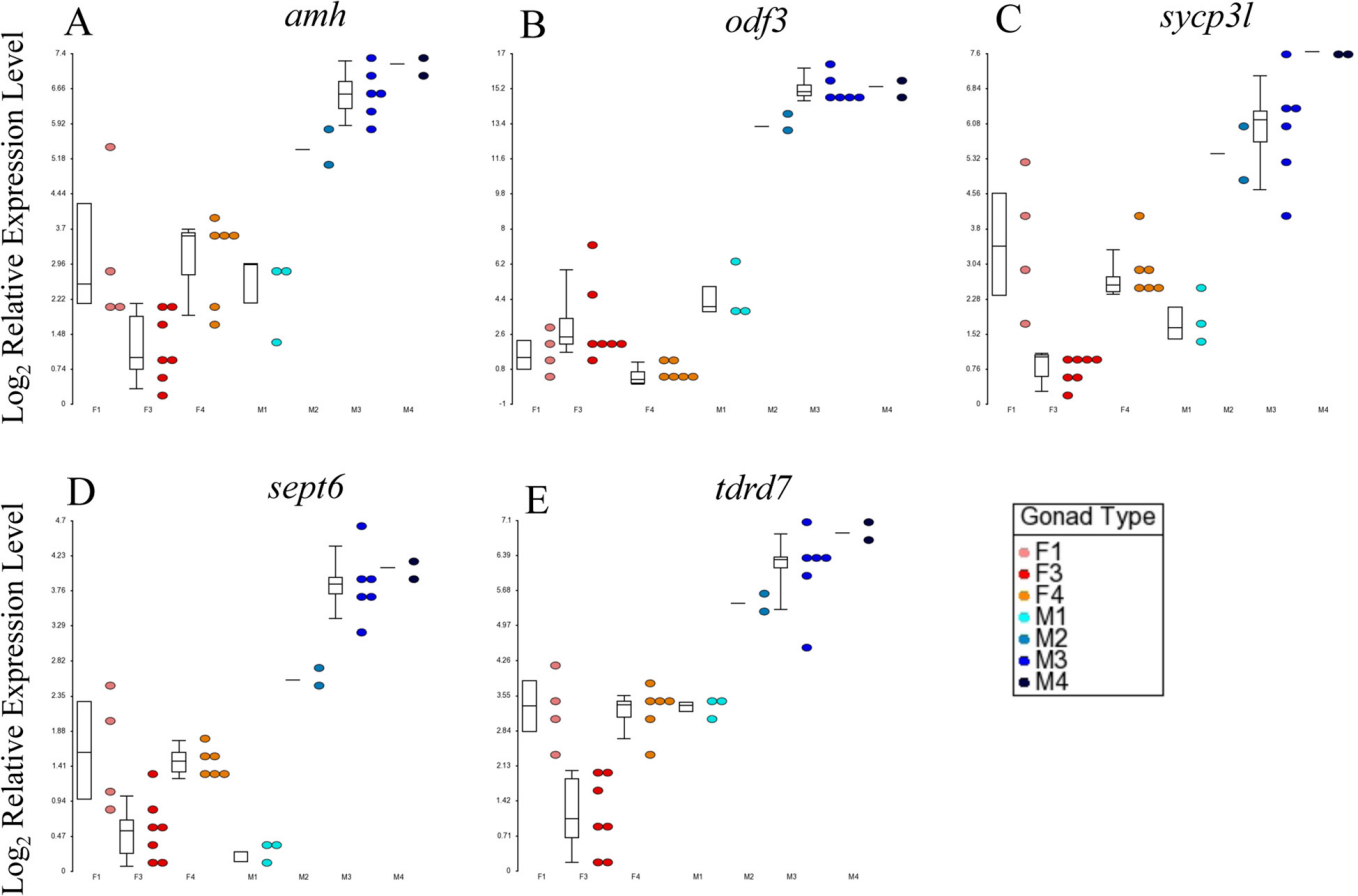
**M1 testes of the Asian seabass showed female-like expression levels. ***amh***(A)**, *odf3 ***(B)**, *sycp3l ***(C)**, *sept6 ***(D)** and *tdrd7 ***(E)** genes. Dot plots overlaid with box plots of the relative log_2_ gene expression values are shown (reference genes: *rpl8*, *ef1a* and *ubq*). The M1 testes are labeled with cyan dots.

Similarly, from the hierarchical clustering map, we can see that, within the testes clade, the inactive M1 testes were clustered in their own sub-clade. M1 testes contain predominately gonia, and these cells are inactive and incapable of spawning. Gene expression analysis showed that these M1 testes have female-like expression levels of the testis-enhanced germ cell markers, *odf3*, *sycp3l*, *septin6* and *tdrd7*, as well as *amh* (Figure 2). This result is likely to be a direct consequence of the lack of spermatids and spermatozoa in the M1 testes because these germ cell markers, *odf3*, *sycp3l* and *septin6*, are involved in the formation of the spermatozoa, as shown in other species, whereas *tdrd7* is associated with male germ cells [29,40,42,44]. In the case of *amh*, it has been demonstrated in the black porgy (*Acanthopagrus schlegeli*) that active testes have higher expression levels than inactive ones, which potentially indicates that a higher level of *amh* might be required to initiate spermatogenesis [82].

### Increased variation of testicular *zp2* expression because of the presence of pre-vitellogenic oocytes in some of the Asian seabass testes

**Figure 3:**
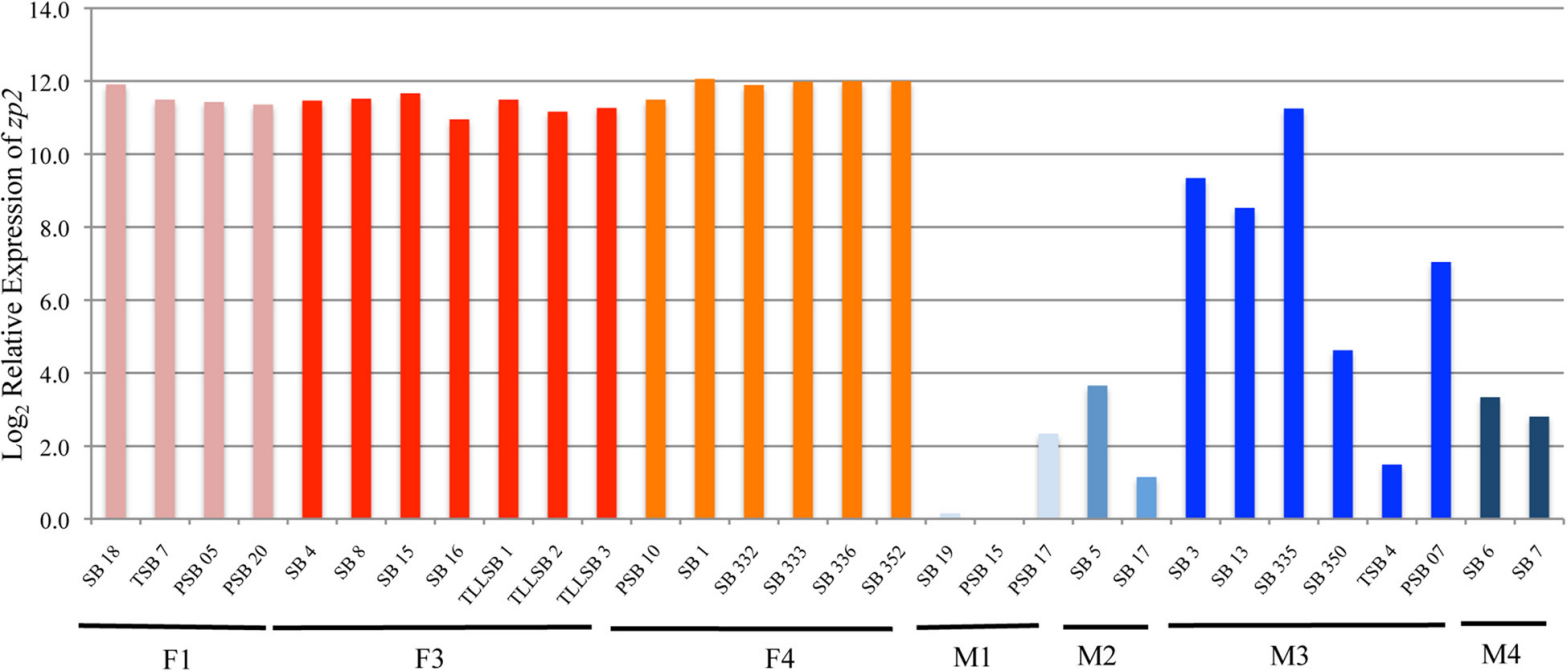
**The expression of *zp2*, which is an oocyte marker, showed a wide variation and an increased level of expression in some M3-type testes in Asian seabass.** The relative log_2_ gene expression values of *zp2* are shown. Female gonads (F1, F3 & F4; pink, red & orange) are on the left, whereas male gonads (M1-M4; light to dark blue) are on the right.

**Figure 4:**
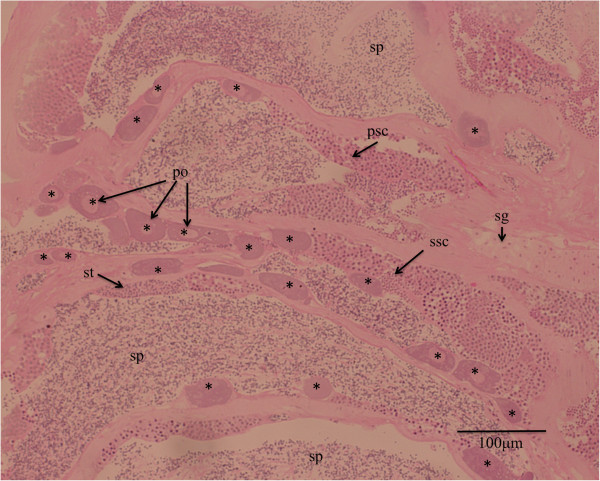
**Pre-vitellogenic oocytes can be found lining the tubular walls in the M3 testis of an Asian seabass individual (SB335).** Abbreviations: po – previtellogenic oocytes; sp – spermatozoa; st – spermatids; ssc – secondary spermatogonia; psc – primary spermatogonia. Pre-vitellogenic oocytes are indicated with asterisks.

Zona pellucida glycoproteins, which are composed of *zp1*, *zp2* and *zp3* in mammals, are found on the extracellular matrix of oocytes and serve as receptors for the binding of sperm; their orthologs were found in fish oocytes [31,83,84]. As expected, in the Asian seabass, *zp2*, which is an oocyte marker, had a 7.1-fold upregulation in the ovaries compared with testes. However, although *zp2* expression showed consistently high expression values across all the female gonads, there was a high variation among the male gonads, with some M3-testes showing increased *zp2* transcript levels compared with the other male gonads (Figure 3). The high *zp2* expression variation may be related to the presence of pre-vitellogenic oocytes that can be found in the histological sections of some M3 testes (Figure 4), which confirms previous descriptions by others [2].

### The sexually dimorphic expression of *cyp11c1* and *esr1* is independent of the gonadal maturation status of gonads

**Figure 5:**
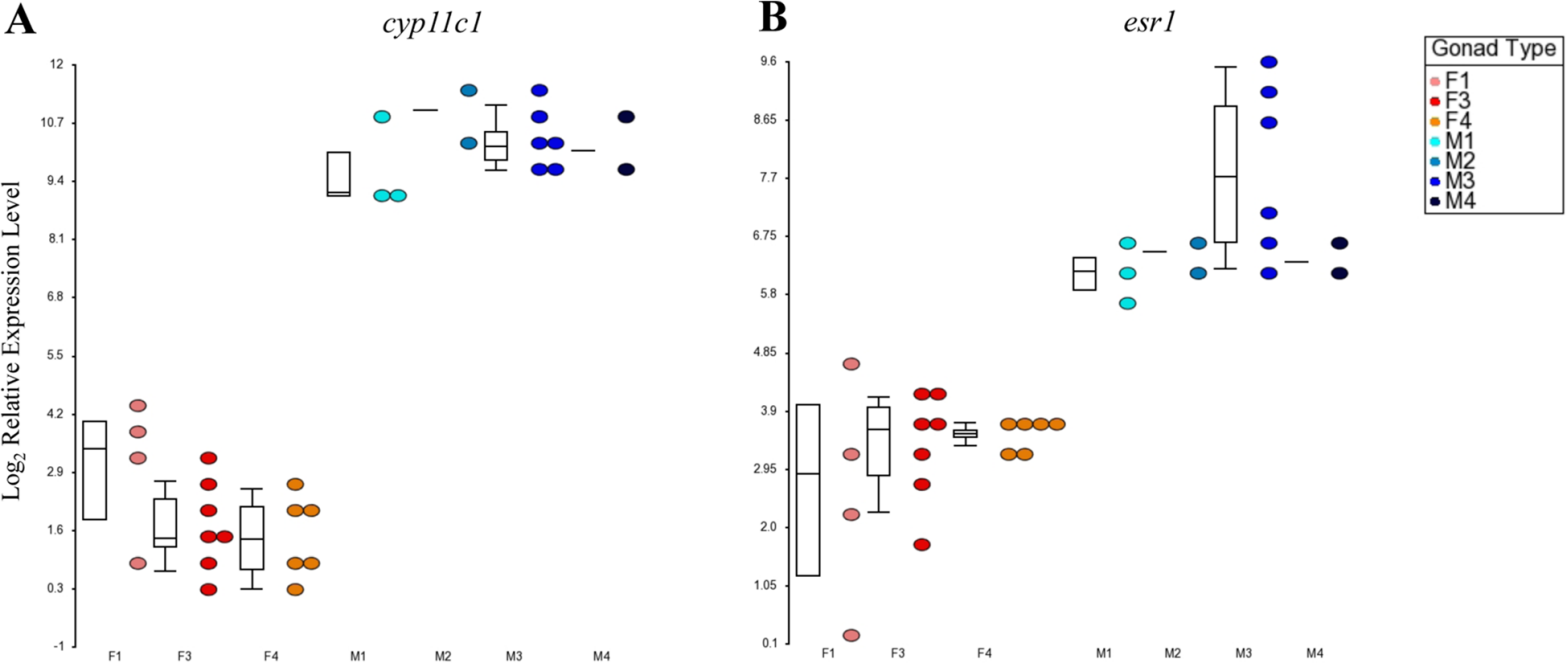
**The expression levels. ***cyp11c1 ***(A)** and *esr1 ***(B)** are distinctly sexually dimorphic between male and female gonads in Asian seabass, regardless of their sexual maturation stage. Dot plots that are overlaid with box plots of the relative log_2_ gene expression values are shown (reference genes: *rpl8*, *ef1a* and *ubq*).

Two of the studied genes, *cyp11c1* and *esr1*, were found to be expressed in a distinctly sexually dimorphic manner between the gonads of the male and female sex, regardless of their sexual maturation stage (Figure 5). Both genes showed testis-enhanced expression, even at the inactive M1 and M2 stages, whereas their expression levels were uniformly low across the F1, F3 and F4 ovaries.

This result indicated that the consistently testis-enhanced expression of *cyp11c1* and *esr1* could be good expression markers for testes and suggested the importance of the two genes in maintaining the male sex of the gonad. Although the upregulation of *esr1* in testes may seem counter-intuitive, given the importance of estrogens in ovary development, estrogen receptors (ER) have been suggested to have a role in the control of testicular function because both *ERa* and *ERb* are expressed in the human testis [85]. Similarly, we do find two paralogs of estrogen receptors in the Asian seabass, but have only analyzed the expression pattern of *esr1* in this study. A higher testicular expression level of *esr1* compared with that of the ovary was also observed in both the medaka and sea bream (*Sparus aurata*) [86-88].

### Zebrafish as a model for reproductive studies in Asian seabass

We propose that the zebrafish can be a model for Asian seabass reproductive studies and possibly even for other sequential hermaphrodites because of the conserved role of several sex-related genes that were described in this study.

**Figure 6:**
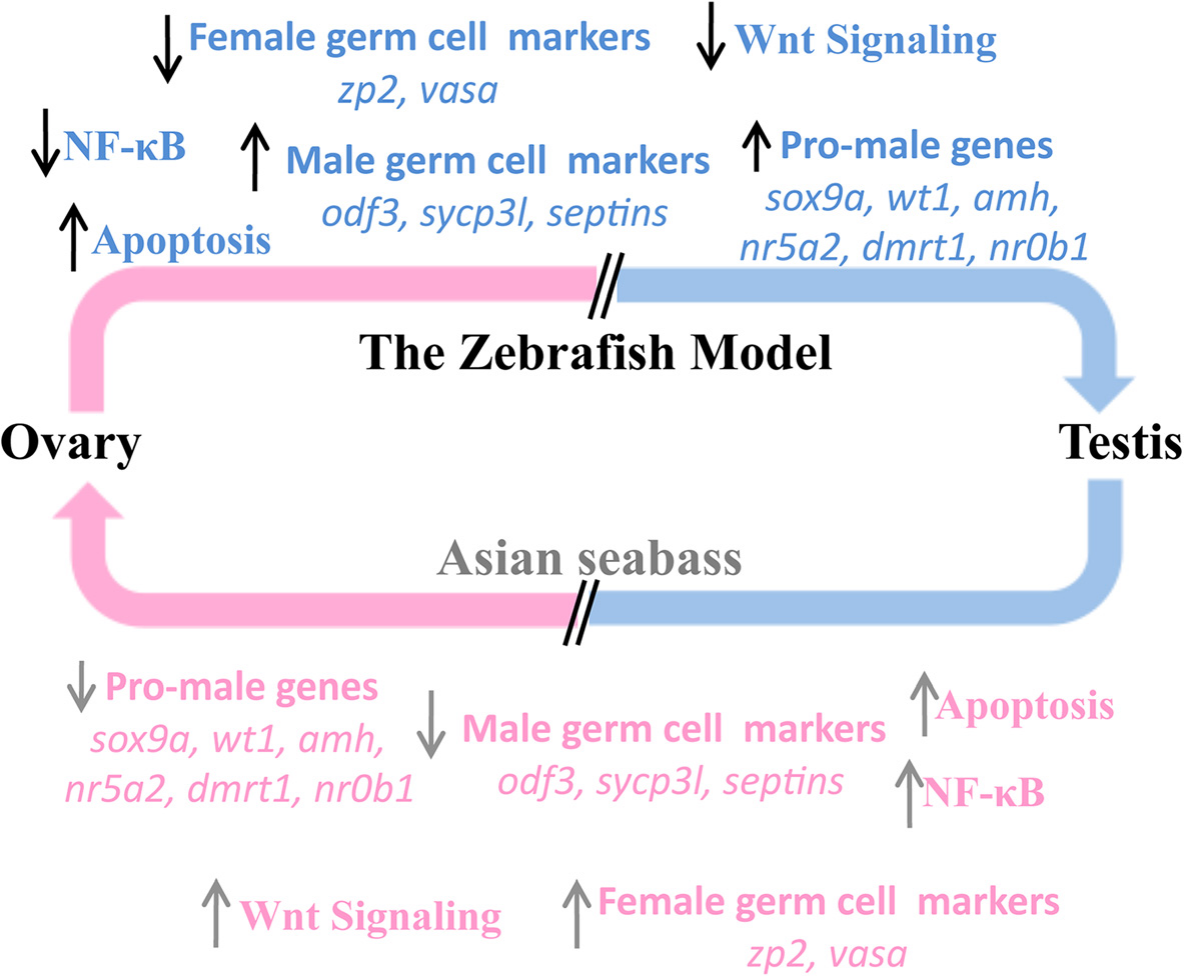
**Working hypothesis for the gonad transformation process.** All zebrafish develop a juvenile ovary before the future males undergo gonadal transformation to form the testis. Several pathways and genes have been known to be involved in this process. Based on our results, we proposed that the same pathways and genes are also involved in the testis-to-ovary transformation process in the Asian seabass, despite the reversal of direction. The Asian seabass system is, hence, a mirror image of the zebrafish model. The arrows show the observed (zebrafish; top - blue) and/or expected (Asian seabass; bottom – pink) up- or downregulation of the genes or pathways during the gonadal transformation process.

All zebrafish develop a juvenile ovary before future males undergo gonadal transformation to form the testis [69,70,89]. Therefore, unlike the Asian seabass, the ovary undergoes gonadal transformation to become the testis, and the process occurs during early differentiation. However, although the zebrafish and Asian seabass systems are developing in the opposite directions (ovary to testis vs. testis to ovary, respectively), we found a largely overlapping set of genes that showed differential expression in the zebrafish and Asian seabass. As such, it seems that the underlying molecular mechanisms that are involved in gonad transformation are conserved in both systems. Therefore, we have developed a working hypothesis for future studies of gonad transformation in the Asian seabass, which are based on the zebrafish model (Figure 6).

In this working model, genes or pathways that are involved in the zebrafish ovary-to-testis transformation are simply “reflected” during Asian seabass testis-to-ovary transformation, in the sense that the same genes and pathways are also involved, except their expression changes in the opposite direction. For example, if a gene or pathway is upregulated during zebrafish ovary-to-testis transformation, then the corresponding ortholog should be downregulated during seabass testis-to-ovary transformation and vice-versa. (Note here that apoptotic genes might be an exception because apoptosis is expected to be higher in the gonad type that will undergo the transformation compared with the other type that will be produced because of the transformation.) Hence, future studies of gonad transformation in Asian seabass can take reference from the well-studied zebrafish model because of the conservation of expression patterns of several genes between the two systems.

## Conclusions

We have generated a platform that allowed us to look at a moderate set of sex-related genes, which were carefully chosen based on earlier data from other species. Our study is the first to report such mid-throughput molecular data regarding gonad maturation in the Asian seabass. The results in our study show that several genes that are known to be involved in reproductive functions are also conserved in their gene expression pattern in the Asian seabass. Our data also indicate that the process of gonad maturation has high individual variability and complexity. The expression patterns of our custom gene set also reflected interesting aspects of the Asian seabass gonads and provided new insights into their sexual maturation and development. In the future, sequencing the complete transcriptome of the species and the use of a whole-transcriptome expression microarray (or RNAseq), together with the working hypothesis that was developed using the zebrafish model, will help us further understand the molecular mechanisms that are involved in gonad transformation and also help us draw parallels with other systems. The use of early undifferentiated and transforming gonads in future studies would also be desired to more accurately identify the genes or pathways that are involved in testis and ovary differentiation, respectively.

## Competing interests

The authors declare that they have no competing interests.

## Authors’ contributions

PR cloned the sex-related sequences, designed the qPCR primers, extracted RNA and performed the qPCR array experiment. JHJ collected and classified the gonad samples, generated RNA samples for next-generation sequencing, assembled the transcriptome, performed the data analysis and drafted the manuscript; WCL participated in the sample collection and performed the qPCR; LO conceived and supervised the work and helped to draft the manuscript. All authors read, corrected and approved the final manuscript.

## Supplementary Material

Additional file 1: Table S1 Gene symbols, with corresponding NCBI accession numbers, and full names of the genes that were analyzed. The identification of the genes that were obtained through this study was performed by running the BLAST algorithm (blastn) in NCBI against the NCBI RefSeq database. Click here for file

Additional file 2: Figure S1 Ovarian and testicular maturation stages in the Asian seabass that were obtained for this study. The classification of the sexual maturation stages was based on Guiguen *et al.*, *Environ Biol Fishes* 1994, 39(3):231–247. Abbreviations: po – pre-vitellogenic oocytes; vi – vitellogenic oocytes; ca – cortical alveolus oocytes; ao – atretic oocytes; g – gonia; st – spermatids; sc – spermatogonia; sp – spermatozoa; r.sp – residual spermatozoa. Click here for file

Additional file 3: Table S2 List of degenerate primer sequences and annealing temperatures that were used in cloning of Asian seabass cDNAs. Click here for file

Additional file 4: Table S3 The list of primer sequences that were used. Click here for file

Additional file 5: Figure S2 Stability values of the candidate reference genes that were obtained using the algorithm GeNorm. The use of three reference genes was optimal for normalization (A), and *rpl8*, *ef1a* and *ubq* had the highest gene expression stability (B). Click here for file

Additional file 6: Table S4 The list of differentially expressed genes between Asian seabass F3 and F4 ovaries was optimal for normalization (A), and *rpl8*, *ef1a* and *ubq* had the highest gene expression stability (B). Click here for file
