# Supplementary figures and images for: Small-scale transcriptomics reveals differences among gonadal stages in Asian seabass (Lates calcarifer)
Source: Reprod Biol Endocrinol. 2014 Jan 9;12:5. doi: 10.1186/1477-7827-12-5 (PMC3896769; doi:10.1186/1477-7827-12-5)

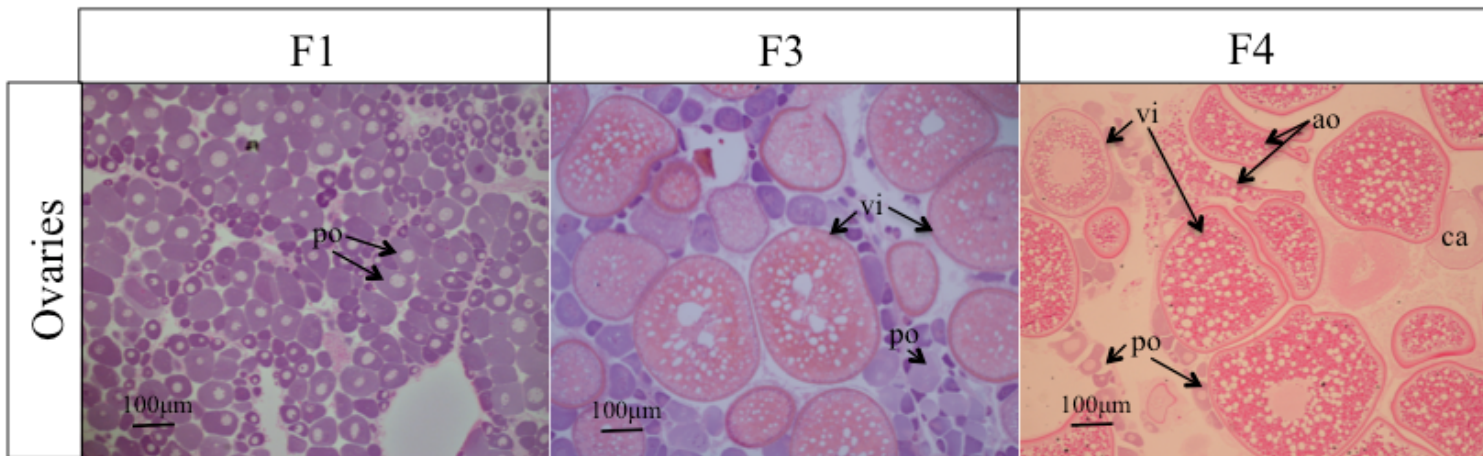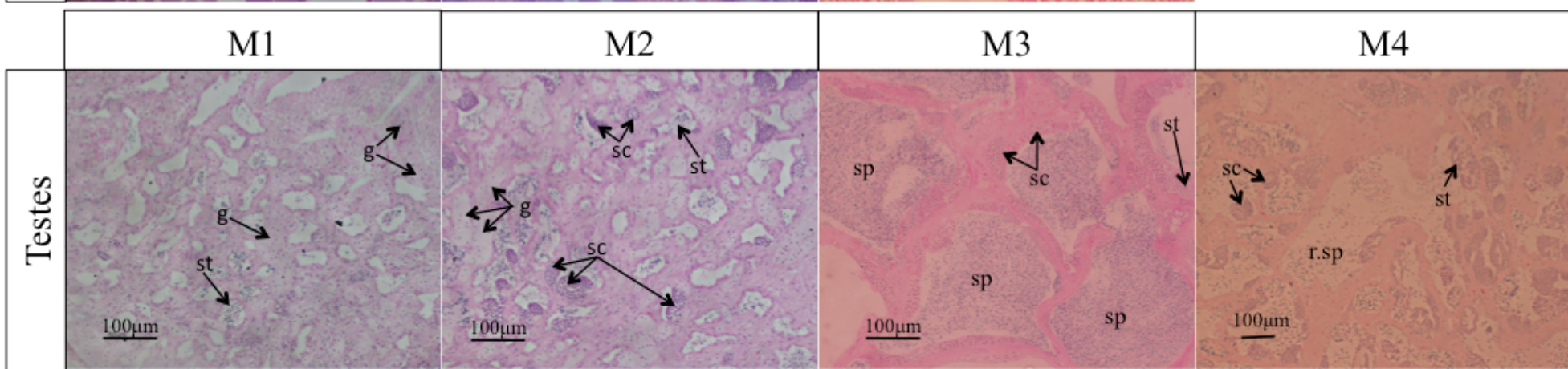

Supplement: Additional file 2: Figure S1 — Ovarian and testicular maturation stages in the Asian seabass that were obtained for this study. The classification of the sexual maturation stages was based on Guiguen et al., Environ Biol Fishes 1994, 39(3):231–247. Abbreviations: po – pre-vitellogenic oocytes; vi – vitellogenic oocytes; ca – cortical alveolus oocytes; ao – atretic oocytes; g – gonia; st – spermatids; sc – spermatogonia; sp – spermatozoa; r.sp – residual spermatozoa. [file 1477-7827-12-5-S2.pdf]
