# Supplementary material for: Small-scale transcriptomics reveals differences among gonadal stages in Asian seabass (Lates calcarifer)
Source: Reprod Biol Endocrinol. 2014 Jan 9;12:5. doi: 10.1186/1477-7827-12-5 (PMC3896769; doi:10.1186/1477-7827-12-5)
Supplement: Additional file 5: Figure S2 — Stability values of the candidate reference genes that were obtained using the algorithm GeNorm. The use of three reference genes was optimal for normalization (A), and rpl8, ef1a and ubq had the highest gene expression stability (B). [file 1477-7827-12-5-S5.pdf]

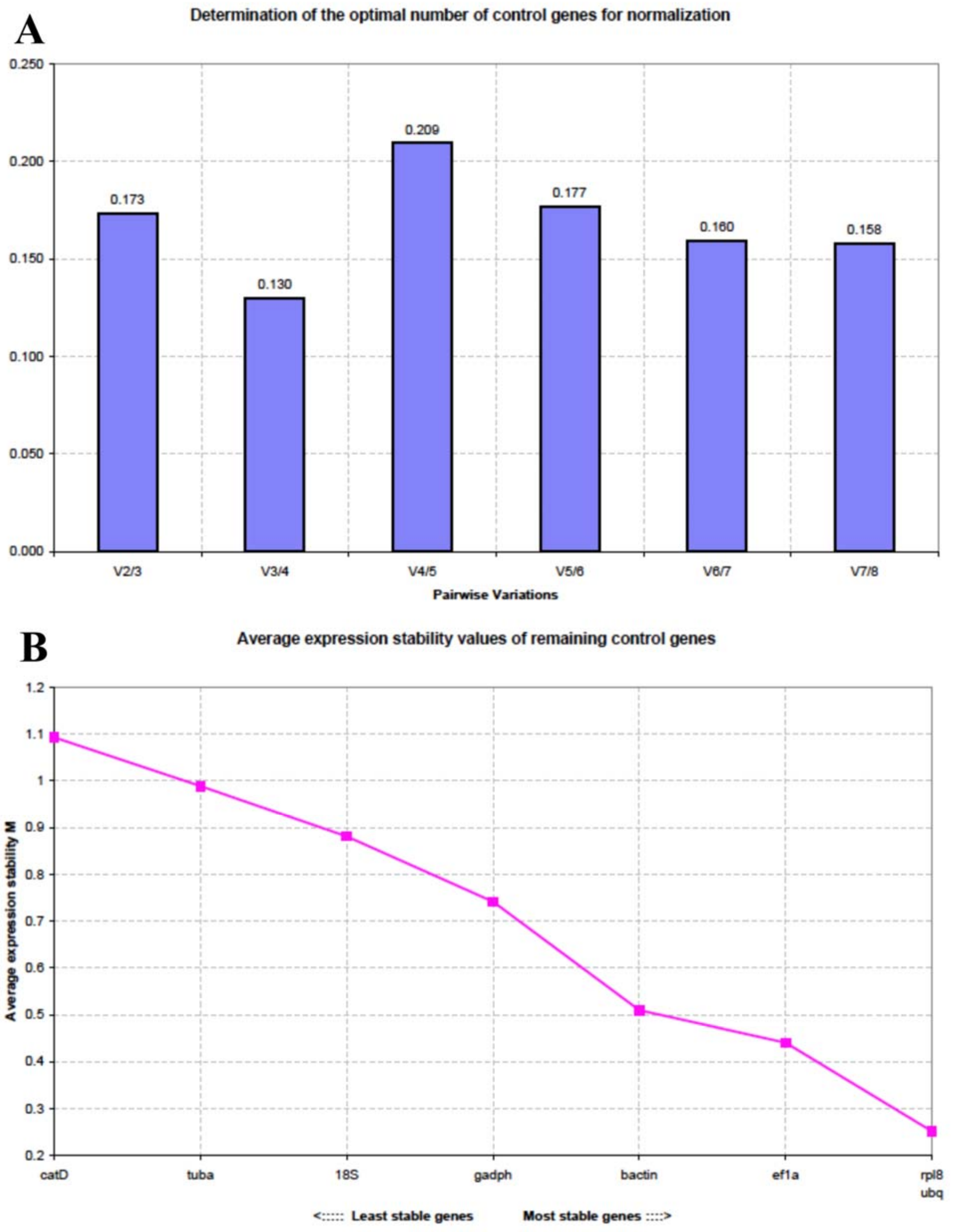

A
Determination of the optimal number of control genes for normalization
0.250
0.200
0.150
0.100
0.050
0.000
0.173
0.130
0.209
0.177
0.160
0.158
V2/3
V3/4
V4/5
V5/6
V6/7
V7/8
Pairwise Variations
B
Average expression stability values of remaining control genes
Average expression stability M
1.2
1.1
1
0.9
0.8
0.7
0.6
0.5
0.4
0.3
0.2
catD
tuba
18S
gadph
bactin
ef1a
rpl8
ubq
<::::: Least stable genes
Most stable genes ::::>
